# Development and Inter-Rater Reliability of the Liverpool Adverse Drug Reaction Causality Assessment Tool

**DOI:** 10.1371/journal.pone.0028096

**Published:** 2011-12-14

**Authors:** Ruairi M. Gallagher, Jamie J. Kirkham, Jennifer R. Mason, Kim A. Bird, Paula R. Williamson, Anthony J. Nunn, Mark A. Turner, Rosalind L. Smyth, Munir Pirmohamed

**Affiliations:** 1 Institute of Child Health, Department of Women's and Children's Health, University of Liverpool, Liverpool, United Kingdom; 2 Department of Biostatistics, University of Liverpool, Liverpool, United Kingdom; 3 Research and Development, Alder Hey Children's NHS Foundation Trust, Liverpool, United Kingdom; 4 Pharmacy, Alder Hey Children's NHS Foundation Trust, Liverpool, United Kingdom; 5 Department of Women's and Children's Health, University of Liverpool, Liverpool, United Kingdom; 6 Department of Molecular and Clinical Pharmacology, Institute of Translational Medicine, University of Liverpool, Liverpool, United Kingdom; Bremen Institute of Preventive Research and Social Medicine, Germany

## Abstract

**Aim:**

To develop and test a new adverse drug reaction (ADR) causality assessment tool (CAT).

**Methods:**

A comparison between seven assessors of a new CAT, formulated by an expert focus group, compared with the Naranjo CAT in 80 cases from a prospective observational study and 37 published ADR case reports (819 causality assessments in total).

**Main Outcome Measures:**

**** Utilisation of causality categories, measure of disagreements, inter-rater reliability (IRR).

**Results:**

The Liverpool ADR CAT, using 40 cases from an observational study, showed causality categories of 1 unlikely, 62 possible, 92 probable and 125 definite (1, 62, 92, 125) and ‘moderate’ IRR (kappa 0.48), compared to Naranjo (0, 100, 172, 8) with ‘moderate’ IRR (kappa 0.45). In a further 40 cases, the Liverpool tool (0, 66, 81, 133) showed ‘good’ IRR (kappa 0.6) while Naranjo (1, 90, 185, 4) remained ‘moderate’.

**Conclusion:**

The Liverpool tool assigns the full range of causality categories and shows good IRR. Further assessment by different investigators in different settings is needed to fully assess the utility of this tool.

## Introduction

Adverse drug reactions are a frequent source of morbidity and mortality [1], [2]. Causality assessment of ADRs may be undertaken by clinicians, academics, pharmaceutical industry, regulators and in different settings, including clinical trials [3], [4], [5], [6]. At an individual level, health care providers assess causality informally when dealing with ADRs in patients to make decisions regarding therapy. Regulatory authorities assess spontaneous ADR reports [4], [5] where causality assessment can help in signal detection and aid in risk-benefit decisions regarding medicines [7], [8].

An early paper by Sir Bradford Hill [9], describing minimum criteria for establishing causality of adverse events, pre-dates the earliest attempts to formulate ADR causality assessment tools. Bradford Hill set out criteria for establishing causality which included assessment of strength of the association, consistency of the association, specificity, temporal relationship, biological gradient (dose response), biological plausibility, coherence, experimental evidence, and reasoning by analogy. Although these criteria were not meant for ADRs, the elements have been adapted in ADR causality tools. Indeed, attempts to formalise causality assessment of ADRs into structured assessment tools have been ongoing for more than 30 years [10], [11]. It is known that assessing ADR likelihood without a structure can lead to wide disagreements between assessors [12]. These disagreements may be the result of differing clinical backgrounds, specialties and experience. The causality tools thus aim to limit disagreement between assessors of ADR cases as to the likelihood that a reaction is related to a particular medication taken by the patient. A large number of causality tools have been developed ranging from the simple to the complex, but none have gained universal acceptance [13].

One of the most widely used causality assessment tools is the Naranjo tool [10]. This is a simple 10-item questionnaire that classifies the likelihood that a reaction is related to a drug using concepts such as timing, plausibility/evidence, de-challenge and re-challenge/previous exposure. Each element of the questionnaire is weighted and the total score used to categorise the event into unlikely, possible, probable and definite. The tool was developed 30 years ago by adult pharmacologists/physicians and psychiatrists. Published case reports were used to validate the reliability of the tool in assessing causality. It has subsequently been widely used, including recently in two prospective observational studies of ADRs causing hospital admission and occurring in hospital in-patients [14], [15]. However, the reliability of the Naranjo tool has been questioned by a number of investigators [3], [8], [16], [17], [18].

While undertaking a prospective observational study of ADRs in children (in preparation), we found several difficulties with using the Naranjo tool. When assessing this heterogeneous mix of potential ADR cases, the investigators found some questions were not appropriate, leading to many answers being categorised as “unknown”. This led to lack of sensitivity as the overall score obtained for each causality assessment may be artificially lowered, which in turn underestimates the likelihood of an ADR. The investigators encountered several cases which were unanimously thought to be definite ADRs (e.g. repeated episodes of febrile neutropenia during oncological chemotherapy) but which did not reach the threshold for definite using the published Naranjo tool. Moreover, the weighting for each question and the ADR classification scoring boundaries used in the Naranjo tool were not justified in the original publication, or subsequently. Therefore, we undertook to develop a causality assessment tool that would overcome some of these issues, while at the same time (a) making it as easy, or easier, to use than the Naranjo tool; and (b) ensuring that the basic principles of assessing causality as defined by Bradford Hill were maintained.

## Methods

Each of seven investigators (RG, JM, KB, MP, TN, RS, MT) independently assessed the first 40 consecutive case reports from a study of suspected ADRs causing hospital admission (ADRIC Study 1 – adverse drug reactions in children available at http://www.adric.org.uk/) using the Naranjo tool. The first 40 cases assessed using Naranjo were reviewed in terms of the results of the pair-wise agreements between the seven investigators. The cases where major discrepancies occurred, that is, where the range of causality probability differed by more than one category (e.g. possible and definite), and the cases where close to half of the raters differed from the others by one category were identified. The questions within the Naranjo tool which caused the discrepancies were identified and reviewed.

Each question in the Naranjo tool was reviewed by the investigators at a consensus meeting to assess whether it was appropriate to incorporate, discard or integrate with other questions into a new, more appropriate, causality tool (Table 1). A new causality tool was drawn up and modified through a consensus approach between the seven investigators. The format of the new tool was an algorithm, or flowchart, with dichotomous responses to each decision followed by routing to further, specific questions, rather than the weighted responses used in the Naranjo tool.

**Table 1:** Decisions made about questions within the Naranjo tool.

No.	Naranjo tool questions	Yes	No	Don't know	Outcome for Liverpool Tool
Q1	Are there previous *conclusive* reports on this reaction?	+1	0	0	**Retained** – knowledge of previous reports can be important when assessing if an adverse event is due to drug or disease.
Q2	Did the adverse event appear after the suspected drug was administered?	+2	−1	0	**Modified** – timing of event in relation to drug exposure is important when determining causality.
Q3	Did the adverse reaction improve when the drug was discontinued or *a specific* antagonist was administered?	+1	0	0	**Modified** – Knowledge of de-challenge, if available, may provide further evidence as to causality of an event. However, an event may have long-lasting sequelae. A new question was added to the Liverpool tool to cover this possibility.
Q4	Did the adverse reaction reappear after the drug was readministered?	+2	−1	0	**Combined** – Knowledge of re-challenge, if available, may add to the level of certainty regarding causality assessment. This question is combined with Naranjo Q8 regarding dose-response relationship to increasing dose. This can also provide evidence to support or refute causality.
Q5	Are there alternative causes (other than the drug) that could on their own have caused the reaction?	−1	+2	0	**Modified** – This question is replaced within the Liverpool tool by a question involving likelihood of alternative cause, with an option to answer ‘unsure’ (which prompts the user to seek further evidence of the reaction). Naranjo Q5 is worded such that it is difficult to answer No.
Q6	Did the reaction reappear when a placebo was given?	−1	+1	0	**Rejected** – With the exception of clinical trials, placebo use is not common practice and this question is no longer relevant.
Q7	Was the drug detected in the blood (or other fluids) in concentrations known to be toxic?	+1	0	0	**Modified** – Objective evidence of the ADR occurrence will already be taken in to account when the user is deciding whether the event is likely to be drug or disease related. A question in the Liverpool tool asks for objective evidence of likely ADR mechanism. If apparent, this may provide evidence of causality to an assessor.
Q8	Was the reaction more severe when the dose was increased, or less severe when the dose was decreased?	+1	0	0	**Combined** – This question is combined with one addressing de-challenge in the Liverpool tool. The answer to this question may be important in establishing if there is a dose-response relationship between drug and adverse event.
Q9	Did the patient have a similar reaction to the same or similar drugs in any previous exposure?	+1	0	0	**Modified** – this is included in the Liverpool algorithm, in relation to the same drug(s) only, and given the same weighting as a positive re-challenge. This may provide evidence of susceptibility, and likelihood, of the event being related to a drug.
Q10	Was the adverse event confirmed by any objective evidence?	+1	0	0	**Modified** – see Q7

The new Liverpool ADR causality tool was then used to assess 20 new suspected ADR case reports from our observational study. All cases assessed from the ADRIC study contained a similar level of documentation. The collated causality categories for all seven assessors showed 1 (0.7%) unlikely, 18 (12.9%) possible, 2 (1.4%) probable and 119 (85%) definite. The assessors achieved moderate agreement with a kappa of 0.51 (95% CI 0.19, 0.82). However, there was an inappropriate bias towards the category of definite which was caused by decision paths leading to an answer of definite without the need for a positive re-challenge or previous reaction with exposure to the same drug. The assessment tool was reviewed again, and major discrepancies between scorers identified and each question within the algorithm reviewed to assess usefulness. Questions and decision pathways that caused major discrepancies were then modified. The new assessment tool was then tested on a further 20 case reports; ten from the ADRIC study and ten from an observational study of in-patient ADRs in an adult hospital. Collated causality categories for the ten ADRIC 1 cases showed 0 (0%) unlikely, 24 (34%) possible, 39 (56%) probable and 7 (10%) definite with a kappa of 0.27 (95% CI 0.11, 0.44). Collated causality categories for the ten adult cases showed 0 (0%) unlikely, 13 (19%) possible, 48 (69%) probable and 9 (13%) definite with a kappa of 0.13 (95% CI −0.14, 0.38).

**Figure 1:**
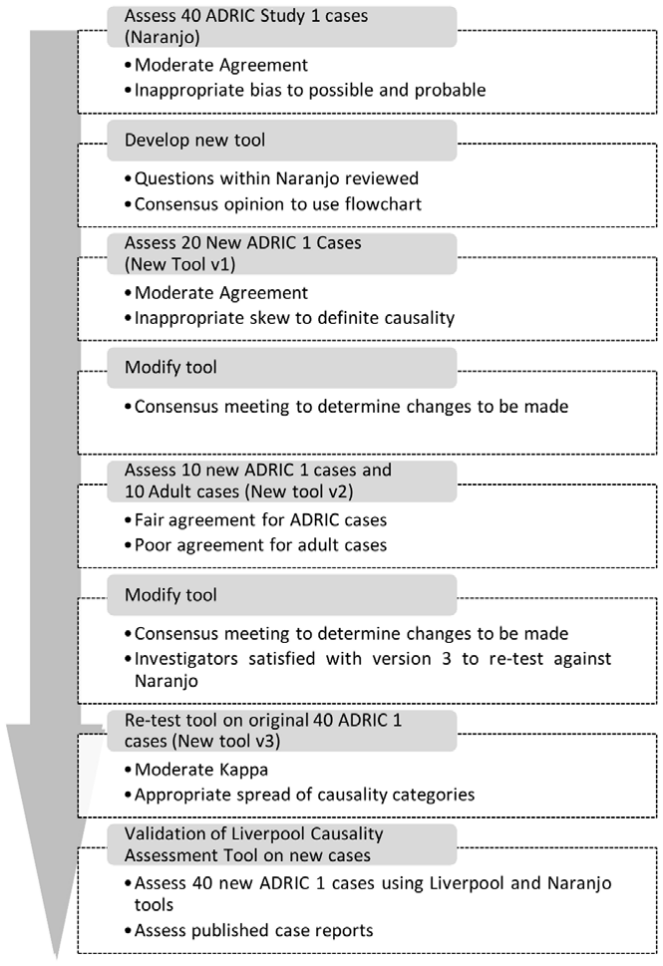
Flowchart of the development of the Liverpool ADR Causality Assessment Tool.

**Figure 2:**
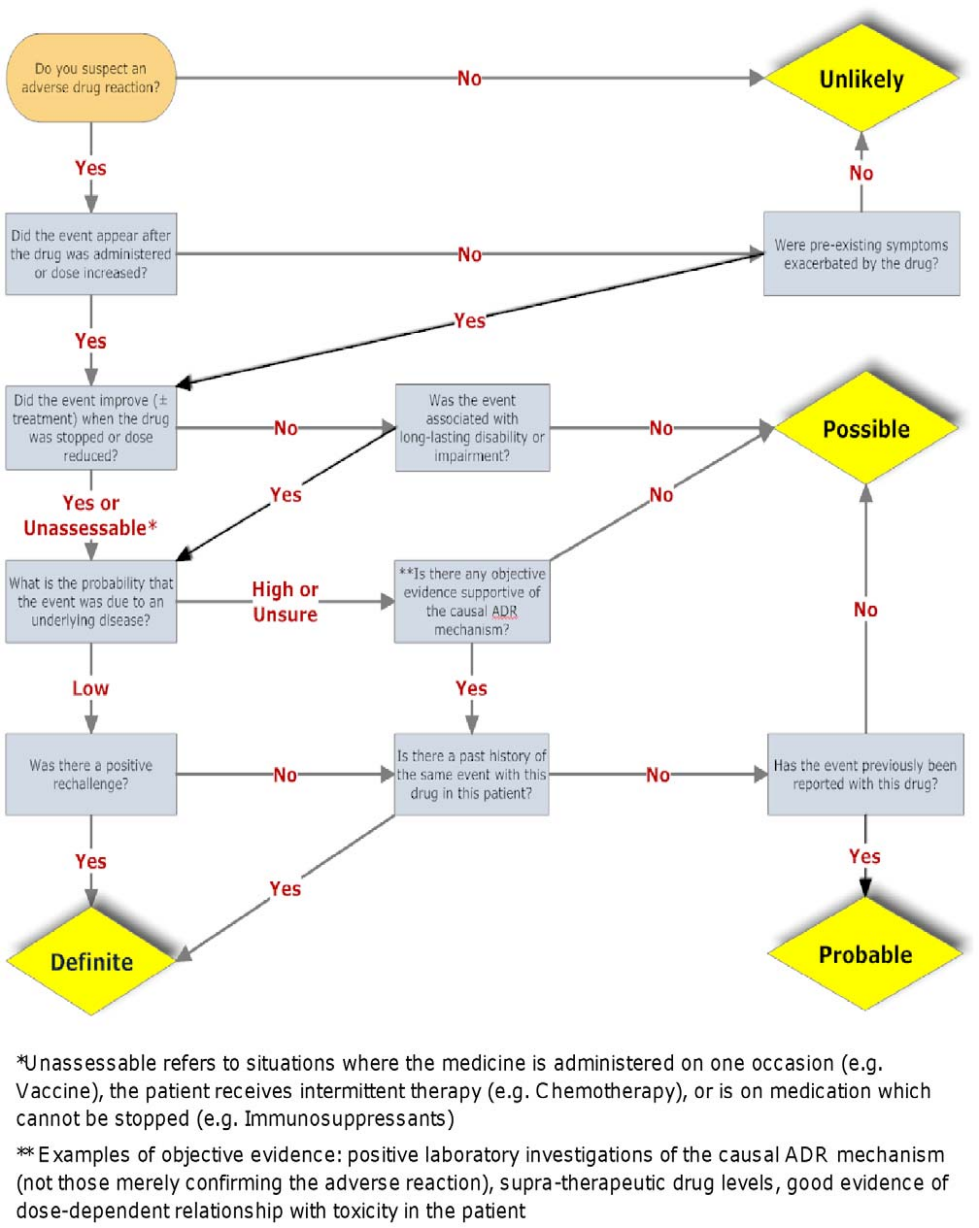
Liverpool ADR causality assessment tool.

The results of these assessments prompted another review of the appropriateness of the tool and questions. A third iteration was used so that the development and evaluation of tool prototypes was based on discussions in which 80 cases were used (Figure 1). After the third iteration the investigators were satisfied with the final version of the new tool (Figure 2) in terms of ease of use, lack of ambiguity, and appropriateness of the causality assignment. This was judged by expert opinion and consensus within the group.

The assessment process for the Liverpool causality assessment tool followed a step-wise procedure:

- The original 40 case reports (case reports of raw clinical data from an observational study) initially assessed with Naranjo were assessed by each of the seven investigators using the new assessment tool to provide a comparison of the inter-rater reliability between the two tools.
- In order to examine the tool using cases other than those collected in our observational study, 37 cases of ADRs were randomly selected from the Annals of Pharmacotherapy (Figure S1) and independently evaluated by the seven assessors using only the new tool. The Annals of Pharmacotherapy requires authors to apply a Naranjo assessment prior to publication of case reports.
- Since the original 40 cases from our observational study had been used in the design of the new tool, a further new set of 40 ADR case reports from our study were then used to compare inter-rater reliability using both the Naranjo and the Liverpool tools.

Categorical scores from both the Naranjo tool and the new tool take the same four point ordinal scale. The inter-rater agreements at each stage of the assessment process were assessed using a linear weighted kappa with 95% confidence intervals for ordered categories. Exact agreement percentages (%EA) were computed to measure the absolute concordances between assessor scores. The percentage of extreme disagreement (%ED), where the causality scores between two raters of the same case are wider than one causality interval apart (e.g. definite for 1 rater and possible for the other), were also computed to measure extreme disagreements between pair-wise rater assessments. To supplement the pair-wise kappas, a global kappa score measuring nominal scale agreement across multiple assessors was calculated with 95% confidence intervals [19]. The global kappa score provides a single statistic to quantify assessor agreement for each set of cases. Kappa values were interpreted according to the guidance from Altman [20]: poor <0.2; fair 0.21–0.40; moderate 0.41–0.60; good 0.61–0.80; and very good 0.81–1.00 agreement.

### Ethics Statement

The observational study of paediatric ADR admissions (ADRIC) was conducted as a service evaluation and this aspect of the study was felt, after discussion with the relevant bodies, not to require an opinion from the Local Research Ethics Committee or the hospital management.

## Results

Assessment of the original 40 consecutive ADR cases by the seven investigators using the Naranjo tool showed collated categorisation of causality scores for all assessors (n = 280 assessments) of 0 (0%) unlikely, 100 (36%) possible, 172 (61%) probable and 8 (3%) definite (Table 2). Exact agreement percentages for the pair-wise comparisons between raters ranged from 43%–93%. Percentage of extreme disagreement (%ED) was 2.5% for four of the twenty-one pair-wise comparisons. There were no extreme disagreements in 17/21 pair-wise comparisons. Pair-wise kappas ranged from 0.27 to 0.86 and the assessors achieved moderate inter-rater reliability with a global kappa of 0.45 (95% CI 0.35–0.54) (Table 3). The same cases assessed using the new Liverpool tool showed collated causality categories of 1 (0.4%) unlikely, 62 (22%) possible, 92 (33%) probable and 125 (45%) definite. Exact agreement percentages ranged from 43–93%. All 21 pair-wise comparisons displayed extreme disagreement with percentages ranging from 5–20%. Pair-wise kappas ranged from 0.27 to 0.84 and the assessors achieved moderate inter-rater reliability with a global kappa score of 0.48 (95% CI 0.42–0.54) (Table 3).

**Table 2:** Causality category assignments of investigators.

		ADRIC Original (N = 40)				Annals of Pharmacotherapy (N = 37)				ADRIC New (N = 40)			
		Unlikely	Possible	Probable	Definite	Unlikely	Possible	Probable	Definite	Unlikely	Possible	Probable	Definite
		n (%)	n (%)	n (%)	n (%)	n (%)	n (%)	n (%)	n (%)	n (%)	n (%)	n (%)	n (%)
**Assessor**	**Tool**												
RG	Naranjo	0 (0.0)	18 (45.0)	22 (55.0)	0 (0.0)	NA	NA	NA	NA	0 (0.0)	18 (45.0)	21 (52.5)	1 (2.5)
	Liverpool	0 (0.0)	7 (17.5)	23 (57.5)	10 (25.0)	0 (0.0)	11 (29.7)	18 (48.7)	8 (21.6)	0 (0.0)	11 (27.5)	12 (30.0)	17 (42.5)
JM	Naranjo	0 (0.0)	17 (42.5)	22 (55.0)	1 (2.5)	NA	NA	NA	NA	0 (0.0)	19 (47.5)	21 (52.5)	0 (0.0)
	Liverpool	0 (0.0)	15 (37.5)	8 (20.0)	17 (42.5)	0 (0.0)	11 (29.7)	20 (54.1)	6 (16.2)	0 (0.0)	14 (35.0)	8 (20.0)	18 (45.0)
KB	Naranjo	0 (0.0)	18 (45.0)	21 (52.5)	1 (2.5)	NA	NA	NA	NA	0 (0.0)	15 (37.5)	25 (62.5)	0 (0.0)
	Liverpool	0 (0.0)	18 (45.0)	4 (10.0)	18 (45.0)	0 (0.0)	12 (32.4)	19 (51.4)	6 (16.2)	0 (0.0)	13 (32.5)	10 (25.0)	17 (42.5)
MT	Naranjo	0 (0.0)	14 (35.0)	24 (60.0)	2 (5.0)	NA	NA	NA	NA	1 (2.5)	9 (22.5)	27 (67.5)	3 (7.5)
	Liverpool	1 (2.5)	5 (12.5)	17 (42.5)	17 (42.5)	0 (0.0)	10 (27.0)	18 (48.7)	9 (24.3)	0 (0.0)	8 (20.0)	9 (22.5)	23 (57.5)
TN	Naranjo	0 (0.0)	10 (25.0)	29 (72.5)	1 (2.5)	NA	NA	NA	NA	0 (0.0)	13 (32.5)	27 (67.5)	0 (0.0)
	Liverpool	0 (0.0)	3 (7.5)	15 (37.5)	22 (55.0)	1 (2.7)	10 (27.0)	20 (54.1)	6 (16.2)	0 (0.0)	8 (20.0)	12 (30.0)	20 (50.0)
MP	Naranjo	0 (0.0)	12 (30.0)	27 (67.5)	1 (2.5)	NA	NA	NA	NA	0 (0.0)	12 (30.0)	28 (70.0)	0 (0.0)
	Liverpool	0 (0.0)	7 (17.5)	12 (30.0)	21 (52.5)	0 (0.0)	10 (27.0)	17 (46.0)	10 (27.0)	0 (0.0)	9 (22.5)	13 (32.5)	18 (45.0)
RS	Naranjo	0 (0.0)	11 (27.5)	27 (67.5)	2 (5.0)	NA	NA	NA	NA	0 (0.0)	4 (10.0)	36 (90.0)	0 (0.0)
	Liverpool	0 (0.0)	7 (17.5)	13 (32.5)	20 (50.0)	0 (0.0)	3 (8.1)	24 (64.9)	10 (27.0)	0 (0.0)	3 (7.5)	17 (42.5)	20 (50.0)
Totals	Naranjo	0 (0.0)	100(35.7)	172 (61.4)	8 (2.9)	0* (0)	5* (13.5)	29* (78.4)	3* (8.1)	1 (0.36)	90 (32.1)	185 (66.1)	4 (1.4)
	Liverpool	1 (0.36)	62 (22.1)	92 (32.9)	125(44.6)	1 (0.39)	67 (25.9)	136 (52.5)	55 (21.2)	0 (0.0)	66 (23.6)	81 (28.9)	133 (47.5)

*It is a journal requirement for authors of case reports in Annals of Pharmacotherapy to complete a Naranjo causality assessment. **Author identification for tables:** Gallagher RM (RG), Mason J (JM), Bird K (KB), Nunn AJ (TN), Turner MA (MT), Smyth RL (RS), Pirmohamed M (MP).

**Table 3:** Naranjo and Liverpool tool assessment of 40 original ADR cases from an observational study.

		Assessor 2							
			RG	JM	KB	MT	TN	MP	RS
Assessor 1	**RG**	%EA/ED		*57.5/0%*	*42.5/0%*	*55.0/0%*	*52.5/0%*	*62.5/0%*	*55.5/0%*
		Kappa (95%CI)		*0.52 (0.27,0.77)*	*0.47 (0.21,0.73)*	*0.44 (0.19,0.69)*	*0.45 (0.21,0.69)*	*0.36 (0.09,0.62)*	*0.29 (0.04,0.54)*
	**JM**	%EA/ED	57.5/5%		***92.5/0%***	*70.0/0%*	*77.5/0%*	*72.5/0%*	*70.0/2.5%*
		Kappa (95%CI)	0.46 (0.26,0.67)		***0.86 (0.71,1.00)***	*0.46 (0.22,0.69)*	*0.56 (0.34,0.78)*	*0.47 (0.19,0.75)*	*0.40 (0.15,0.65)*
	**KB**	%EA/ED	42.5/10%	**75.0/5%**		***77.5/0%***	*70.0/0%*	*70.0/0%*	*77.5/2.5%*
		Kappa (95%CI)	0.28 (0.08,0.49)	**0.69 (0.52,0.87)**		***0.60 (0.39,0.81)***	*0.43 (0.19,0.66)*	*0.43 (0.15,0.71)*	*0.55 (0.32,0.77)*
	**MT**	%EA/ED	55.0/7.5%	**70.0/5%**	57.5/7.5%		*72.5/0%*	*62.5/0%*	*70.0/2.5%*
		Kappa (95%CI)	0.31 (0.06,0.56)	**0.62 (0.45,0.80)**	0.49 (0.31,0.67)		*0.45 (0.20,0.70)*	*0.37 (0.11,0.62)*	*0.48 (0.23,0.73)*
	**TN**	%EA/ED	52.5/7.5%	62.5/15%	52.5/20%	70.0/7.5%		*70.0/0%*	*72.5/2.5%*
		Kappa (95%CI)	0.27 (0.07,0.46)	0.42 (0.21,0.62)	0.30 (0.10,0.50)	0.49 (0.26,0.72)		*0.33 (0.05,0.62)*	*0.35 (0.06,0.63)*
	**MP**	%EA/ED	62.5/5%	**77.5/7.5%**	67.5/12.5%	**80.0/5%**	**80.0/7.5%**		*70.0/0%*
		Kappa (95%CI)	0.47 (0.25,0.69)	**0.68 (0.49,0.86)**	0.54 (0.33,0.74)	**0.69 (0.49,0.89)**	**0.62 (0.39,0.84)**		*0.38 (0.11,0.65)*
	**RS**	%EA/ED	55.5/10%	70.0/12.5%	62.5/15%	**80.0/7.5%**	75.0/10%	**92.5/5%**	
		Kappa (95%CI)	0.30 (0.05,0.55)	0.54 (0.32,0.76)	0.46 (0.24,0.67)	**0.66 (0.44,0.87)**	0.52 (0.27,0.76)	**0.84 (0.66,1.00)**	

%EA/ED and Kappa scores in italics represent Naranjo tool analyses.

%EA/ED and Kappa scores in normal font represent Liverpool ADR causality tool analyses.

Kappa scores outlined in bold demarcate either a good or very good level of agreement.

The 37 randomly selected ADR case reports from the Annals of Pharmacotherapy assessed by the seven investigators using the Liverpool tool showed collated categorisation of causality scores (n = 259 assessments) of 1 (0.4%) unlikely, 67 (26%) possible, 136 (53%) probable and 55 (21%) definite. Exact agreement percentages ranged from 57%–97%. 18/21 pair-wise comparisons between raters showed some extreme disagreement, with the percentage ranging from 5–11%, while three showed no extreme disagreements. Pair-wise kappas ranged from 0.31 to 0.96 and the assessors achieved moderate inter-rater reliability with a global kappa of 0.43 (95% CI 0.34–0.51) (Table 4). These case reports were not assessed by the investigators using the Naranjo tool as The Annals of Pharmacotherapy requires authors to apply a Naranjo assessment prior to publication of case reports in the journal. The collated categorization of the case report author assessments for the 37 cases showed 0 unlikely, 5 (14%) possible, 29 (78%) probable and 3 (8%) definite.

**Table 4:** Liverpool ADR Causality tool assessment of 37 randomly selected published ADR case reports.

		Assessor 2							
			RG	JM	KB	MT	TN	MP	RS
Assessor 1	RG	%EA/ED		62.2/10.8%	64.9/10.8%	**73.0/0%**	56.8/8.1%	59.5/5.4%	67.6/5.4%
		Kappa (95% CI)		0.307 (0.03,0.58)	0.38 (0.10,0.65)	**0.65 (0.44,0.85)**	0.32 (0.05,0.59)	0.41 (0.16,0.66)	0.46 (0.22,0.69)
	JM	%EA/ED			**97.3/0%**	62.2/10.8%	64.9/8.1%	56.8/8.1%	64.9/8.1%
		Kappa (95% CI)			**0.93 (0.82,1.00)**	0.31 (0.04,0.59)	0.34 (0.06,0.61)	0.29 (0.02,0.57)	0.33 (0.09,0.57)
	KB	%EA/ED				59.5/10.8%	67.6/8.1%	59.5/8.1%	62.2/8.1%
		Kappa (95% CI)				0.31 (0.03,0.59)	0.41 (0.13,0.68)	0.36 (0.10,0.63)	0.34 (0.10,0.58)
	MT	%EA/ED					64.9/8.1%	64.9/5.4%	**78.4/5.4%**
		Kappa (95% CI)					0.40 (0.13,0.66)	0.48 (0.23,0.72)	**0.61 (0.38,0.84)**
	TN	%EA/ED						62.2/8.1%	67.6/5.4%
		Kappa (95% CI)						0.38 (0.11,0.64)	0.42 (0.19,0.65)
	MP	%EA/ED							70.3/0%
		Kappa (95% CI)							0.58 (0.38,0.77)
	RS								

Kappa scores outlined in bold demarcate either a good or very good level of agreement.

The 40 newly selected ADR cases assessed by the seven investigators using the Naranjo tool showed collated categorisation of causality scores (n = 280 assessments) of 1 (0.4%) unlikely, 90 (32%) possible, 185 (66%) probable and 4 (1%) definite. Exact agreement percentages ranged from 63%–90%. Percentage of extreme disagreement was 2.5% for four pair-wise comparisons. There were no extreme disagreements in 17/21 comparisons. The pair-wise kappas ranged from 0.19 to 0.81 with moderate inter-rater reliability and global kappa of 0.44 (95% CI 0.33–0.55) (Table 5). The same cases assessed using the Liverpool tool showed collated causality categories of 0 (0%) unlikely, 66 (24%) possible, 81 (29%) probable and 133 (48%) definite. Exact agreement percentages ranged from 65%–88%. Percentage of extreme disagreement ranged from 2.5–7.5% for 14 pair-wise comparisons. There were no extreme disagreements in 7/21 comparisons. Pair-wise kappas ranged from 0.51 to 0.85 and the assessors achieved good inter-rater reliability with a global kappa of 0.60 (95% CI 0.54–0.67) (Table 5).

**Table 5:** Naranjo and Liverpool tool assessment of 40 new ADR cases from an observational study.

		Assessor 2							
			RG	JM	KB	MT	TN	MP	RS
Assessor 1	**RG**	%EA/ED		***90.0/0%***	***80.0/0%***	*70.0/2.5%*	*75.0/0%*	*72.5/0%*	*62.5/0%*
		Kappa (95%CI)		***0.81 (0.64,0.98)***	***0.61 (0.38,0.84)***	*0.46 (0.25,0.66)*	*0.51 (0.26,0.75)*	*0.46 (0.20,0.71)*	*0.23 (0.03,0.42)*
	**JM**	%EA/ED	**70.0/5%**		*75.0/0%*	*67.5/0%*	*80.0/0%*	*77.5/0%*	*62.5/0%*
		Kappa (95%CI)	**0.62 (0.43,0.81)**		*0.49 (0.23,0.76)*	*0.45 (0.25,0.64)*	*0.59 (0.35,0.83)*	*0.54 (0.29,0.79)*	*0.22 (0.02,0.41)*
	**KB**	%EA/ED	**65.0/0%**	**77.5/2.5%**		*70.0/2.5%*	*80.0/0%*	*77.5/0%*	*67.5/0%*
		Kappa (95%CI)	**0.62 (0.44,0.79)**	**0.73 (0.57,0.90)**		*0.40 (0.16,0.63)*	*0.56 (0.29,0.83)*	*0.50 (0.22,0.78)*	*0.19 (−0.06,0.44)*
	**MT**	%EA/ED	**70.0/2.5%**	**75.0/5%**	**75.0/7.5%**		*70.0/2.5%*	*70.0/2.5%*	*72.5/0%*
		Kappa (95%CI)	**0.63 (0.45,0.81)**	**0.70 (0.52,0.88)**	**0.64 (0.45,0.84)**		*0.367 (0.12,0.62)*	*0.40 (0.15,0.65)*	*0.25 (0.003,0.50)*
	**TN**	%EA/ED	**82.5/2.5%**	**77.5/2.5%**	**70.0/2.5%**	**82.5/0%**		*77.5/0%*	*77.5/0%*
		Kappa (95%CI)	**0.77 (0.61,0.93)**	**0.73 (0.57,0.88)**	**0.61 (0.43,0.79)**	**0.79 (0.64,0.93)**		*0.48 (0.18,0.77)*	*0.38 (0.09,0.66)*
	**MP**	%EA/ED	**70.0/2.5%**	**80.0/2.5%**	**72.5/2.5%**	**80.0/0%**	**87.5/0%**		*80.0/0%*
		Kappa (95%CI)	**0.63 (0.44,0.81)**	**0.75 (0.59,0.91)**	**0.64 (0.46,0.82)**	**0.76 (0.61,0.91)**	**0.85 (0.73,0.97)**		*0.41 (0.12,0.71)*
	**RS**	%EA/ED	**70.0/2.5%**	70.0/5%	65.0/5%	**80.0/0%**	**82.5/0%**	**75.0/0%**	
		Kappa (95%CI)	**0.60 (0.42,0.78)**	0.57 (0.40,0.74)	0.50 (0.31,0.69)	**0.73 (0.58,0.88)**	**0.77 (0.62,0.91)**	**0.67 (0.51,0.84)**	

%EA/ED and Kappa scores in italics represent Naranjo tool analyses.

%EA/ED and Kappa scores in normal font represent Liverpool ADR causality tool analyses.

Kappa scores outlined in bold demarcate either a good or very good level of agreement.

## Discussion

A recent systematic review of studies assessing the reliability of causality assessments concluded that “no causality assessment method has shown consistent and reproducible measure of causality.”[3] We are currently undertaking a comprehensive assessment of adverse drug reactions in children [21]. As part of this, we had initially decided to use the Naranjo tool to assess causality in our patients admitted with ADRs, and those who developed ADRs as in-patients. In order to do this, we planned to have assessments conducted independently by seven assessors. Initial assessments revealed some significant issues with the Naranjo tool (as outlined in the introduction above), which led us to develop the Liverpool Causality Assessment Tool.

The development of the Liverpool Causality Assessment Tool involved an iterative process conducted by a multidisciplinary team using raw case data and published case reports. The clinical team included nurses, pharmacists and physicians, including those working with adults and children. Previous experience with formal ADR assessment ranged from minimal to advanced. The assessment team comprised medical statisticians who focused discussion on how to classify cases and monitored progress using standard tools for inter-rater agreement. This approach has the strength of timeliness but the potential weaknesses of “group-think”, in which independent thinking and expression of differences may be lost in the pursuit of group cohesiveness.

We believe that the Liverpool Causality tool has several advantages over the Naranjo tool. First, it performed as well as the Naranjo tool with the first set of cases that were assessed. The inter-rater reliability improved over time with the new tool, whereas the inter-rater reliability when using Naranjo remained similar, despite the fact that there was as much exposure to this tool within the assessing group. The improved inter-rater reliability with the new tool may be explained by increasing experience of its use.

The proportion of exact agreements between assessors was comparable between the two tools for both sets of cases despite the improvement in the global kappa for the new tool. This is because it is difficult to achieve a ‘definite’ category using the Naranjo tool and assessors mainly scored cases as ‘possible’ or ‘probable.’ Therefore, the chances of exact agreement between two assessors of the same case using the Naranjo tool are likely to be falsely elevated compared to the kappa scores which adjust for chance agreement. This paradox has been discussed previously in the literature [22], [23], [24].

The percentage of extreme disagreement between raters was higher for the Liverpool tool, when compared to Naranjo. Due to the difficulty in achieving a ‘definite’ score with Naranjo the chances of finding extreme disagreement, when comparing pair-wise assessments, is likely to be falsely low. The observed percentage of extreme disagreements decreased when using the Liverpool tool from the first set of 40 cases to the last set. This may also be explained by increasing experience of its use.

Second, the inter-rater reliability on assessing published case reports with the new tool was similar to that when we assessed our observational study cases with the Naranjo tool. Five of the seven assessors work in paediatric practice and the published case reports were adult cases. This perhaps provides an indication, albeit indirectly, of the robustness of the tool in assessing a range of case reports, even when used by assessors for cases from unfamiliar clinical settings.

Third, in the Naranjo tool, almost all cases were categorised as possible or probable. With the new tool, the range of categorisations was broader with some cases judged as being definite. A novel aspect of the tool which made this possible was that prior exposure that led to the same ADR, for example during a previous course of chemotherapy, was included and was thus judged as being equivalent to a prospective re-challenge. The high proportion of definite causality assessments can be explained by the fact that our study contained a large number of children with malignancies who had repeated courses of chemotherapy. It is also important to note that the cases were extracted from an observational study of suspected ADRs in children, and thus some case selection had occurred *a priori* making it improbable to record a score of ‘unlikely’ when assessing with either tool.

Fourth, a flow diagram rather than scoring system was used in the new tool for causality assessment and was felt by assessors to be easy to follow and quick to complete. We used a classification approach based on binary decisions (taking account of “don't know” responses). In this case, it is important to ensure that the binary decisions are robust. Once this has been done, then the instrument should be relatively context-independent. A weighted scoring system, such as the Naranjo tool, however will give more influence to some variables than others. A weighting scheme requires the validation of both the items in the tool and the weightings themselves. Ideally, the weightings need to be developed and validated in a context that is similar to the context in which they are applied. Thus a weighting scheme is more likely to be sensitive and specific within a defined context (as long as you have a gold standard) but is more likely to be context-dependent. Thus we would conclude, that for ADRs where many different drugs can cause reactions in different settings, and where the patient's ADR may be assessed by healthcare professionals from a variety of backgrounds, it is more important to develop a tool that is context-independent.

Not unexpectedly, we were unable to achieve complete agreement about causality assessment for a minority of suspected ADRs. Most likely, this reflects underlying uncertainty arising from issues such as the perceived likelihood of alternative explanations. These perceptions will vary between raters depending on their experience or professional backgrounds.

In summary, we present a new causality assessment tool, developed by a multi-disciplinary team, which performed better than the Naranjo tool. We believe the new tool to be practicable and likely to be acceptable for use by healthcare staff in assessing ADRs. We have undertaken a validation of the tool, with a total of 819 causality assessments by seven investigators, using investigators within our ADRIC research programme. Although this validation is equivalent, if not better, than that undertaken for many other tools [10], [25], [26], one limitation is that the increase in IRR for the second set of 40 case reports using the new tool remains unexplained. We plan to investigate this using external validation in a randomised clinical trial. Another limitation is that the validation has been undertaken internally and not independently by other investigators. However, we feel that the tool shows promise, and by publishing it, we hope it will allow other investigators to undertake independent assessments of the usefulness of this tool in other populations (e.g. using data from adult or elderly care settings), not only for spontaneous reports but also for adverse events occurring within trials.

## Supporting Information

Figure S1 **Annals of Pharmacotherapy published adverse drug reaction case reports assessed using the Liverpool ADR Causality Assessment Tool.**
(DOC)
