# Supplementary material for: Development and Inter-Rater Reliability of the Liverpool Adverse Drug Reaction Causality Assessment Tool
Source: PLoS One. 2011 Dec 14;6(12):e28096. doi: 10.1371/journal.pone.0028096 (PMC3237416; doi:10.1371/journal.pone.0028096)
Supplement: Figure S1 — Annals of Pharmacotherapy published adverse drug reaction case reports assessed using the Liverpool ADR Causality Assessment Tool. (DOC) [file pone.0028096.s001.doc]

Figure S1. Published adverse drug reaction case reports assessed using the Liverpool ADR Causality Assessment Tool

Dana G Carroll, and Lamont E Cavanagh. Drug-Induced Lupus Associated with Synthetic Conjugated Estrogens. *Ann Pharmacother*; **41**: 702-706.

Miroslav Cuturic, and Ruth K Abramson. Acute Hyperammonemic Coma with Chronic Valproic Acid Therapy. *Ann Pharmacother*; **39**: 2119-2122.

Raul Noguera-Pons, Joaquín Borrás-Blasco, Isabel Romero-Crespo, Rosa Antón-Torres, Andrés Navarro-Ruiz, and Jose Antonio González-Ferrandez. Optic Neuritis with Concurrent Etanercept and Isoniazid Therapy. *Ann Pharmacother*; **39**: 2131-2134.

Shu-Hwa Hsiao, Li-Hsiang Liao, Pin-Nan Cheng, and Ta-Jen Wu. Hepatotoxicity Associated with Acarbose Therapy. *Ann Pharmacother*; **40**: 151-154.

Allan L Bernstein, and Ann Werlin. Pseudodementia Associated with Use of Ibuprofen. *Ann Pharmacother*; **37**: 80-82.

Tracy D Watson, Jennifer E Stark, and Kimi S Vesta. Pantoprazole-Induced Thrombocytopenia.
*Ann Pharmacother*; **40**: 758-761.

Konstantinos N Fountoulakis, Melina Siamouli, Sotiris Kantartzis, Panagiotis Panagiotidis, Apostolos Iacovides, and George St Kaprinis. Acute Dystonia with Low-Dosage Aripiprazole in Tourette's Disorder. *Ann Pharmacother*; **40**: 775-777.

Harindra Karunatilake, and Nicholas A Buckley. Serotonin Syndrome Induced by Fluvoxamine and Oxycodone. *Ann Pharmacother*; **40**: 155-157.

Todd J Kowalski, Michael J Henry, and Jonathan A Zlabek. Furazolidone-Induced Pulmonary Hypersensitivity. *Ann Pharmacother*; **39**: 377-379.

Abu Fanne Rami, Daniel Barkan, Dror Mevorach, Eran Leitersdorf, and Yoseph Caraco. Clozapine-Induced Systemic Lupus Erythematosus. *Ann Pharmacother*; **40**: 983-985.

Mary E Gordinier, and Don S Dizon. Dyspnea During Thalidomide Treatment for Advanced Ovarian Cancer. *Ann Pharmacother*; **39**: 962-965.

Moustafa HM El-Naggar, Ahmed Helmy, Mahmoud Moawad, Mohamed Al-Omary, Yusuf Al-Kadhi, and Bassil Habib. Late-Onset Rosiglitazone-Associated Acute Liver Failure in a Patient with Hodgkin's Lymphoma. *Ann Pharmacother*; **42**: 713-718.

Kalpana P Padala, Prasad R Padala, and Jane F Potter. Simvastatin-Induced Decline in Cognition. *Ann Pharmacother*; **40**: 1880-1883.

Joaquín Borrás-Blasco, Claudia Nuñez-Cornejo, Antonio Gracia-Perez, J Dolores Rosique-Robles, MD Elvira Casterá, Enrique Viosca, and F Javier Abad. Parapharyngeal Abscess in a Patient Receiving Etanercept. *Ann Pharmacother*; **41**: 341-344.

Sunny A Linnebur, and William H Hiatt. Probable Statin-Induced Testicular Pain. *Ann Pharmacother*; **41**: 138-142.

Sarah A Nisly, Shaunta' M Ray, and Robert A Moye. Tobramycin-Induced Hepatotoxicity. *Ann Pharmacother*; **41**: 2061-2065.

Seema Jabeen, Stephanie I Polli, and David R Gerber. Acute Respiratory Failure with a Single Dose of Quetiapine Fumarate. *Ann Pharmacother*; **40**: 559-562.

Alice YY Cheng, and I George Fantus. Thiazolidinedione-Induced Congestive Heart Failure. *Ann Pharmacother*; **38**: 817-820.

Jen-Wei Chou, Cheng-Ju Yu, Po-Heng Chuang, Hsueh-Chou Lai, Chang-Hu Hsu, Ken-Sheng Cheng, Cheng-Yuan Peng, and I-Ping Chiang. Successful Treatment of Fosinopril-Induced Severe Cholestatic Jaundice with Plasma Exchange. *Ann Pharmacother*; **42**: 1887-1892.

Murugan Raghavan, Mark A Mazer, and David J Brink. Severe Hypersensitivity Pneumonitis Associated with Anagrelide. *Ann Pharmacother*; **37**: 1228-1231.

Sheau-Chiou Chao, Chao-Chun Yang, and Julia Yu-Yun Lee. Hypersensitivity Syndrome and Pure Red Cell Aplasia Following Allopurinol Therapy in a Patient with Chronic Kidney Disease. *Ann Pharmacother*; **39**: 1552-1556.

C Phu Pham, Peter W de Feiter, P Hugo M van der Kuy, and Walther NKA van Mook. Long QTc Interval and Torsade de Pointes Caused by Fluconazole. *Ann Pharmacother*; **40**: 1456-1461.

Paola L Minciullo, Antonella Saija, Domenica Bonanno, Edoardo Ferlazzo, and Sebastiano Gangemi. Montelukast-Induced Generalized Urticaria. *Ann Pharmacother*; **38**: 999-1001.

Brian K Irons, and Ashwani Kumar. Valsartan-Induced Angioedema. *Ann Pharmacother*; **37**: 1024-1027.

Shyam D Karki, and Gule-Rana Masood. Combination Risperidone and SSRI–Induced Serotonin Syndrome. *Ann Pharmacother*; **37**: 388-391.

Karen E Moeller, and Susan C Touma. Prolonged Thrombocytopenia Associated with Isotretinoin. *Ann Pharmacother*; **37**: 1622-1624.

Diane Nykamp, and Erin E Winter. Olmesartan Medoxomil-Induced Angioedema. *Ann Pharmacother*; **41**: 518-520.

Marisel Segarra-Newnham, and Shari S Tagoff. Probable Vancomycin-Induced Neutropenia. *Ann Pharmacother*; **38**: 1855-1859.

Kimi S Vesta, and Patrick J Medina. Valproic Acid–Induced Neutropenia. *Ann Pharmacother*; **37**: 819-821.

A Scott Mathis, Vicky Chan, Margaret Gryszkiewicz, Robert T Adamson, and Gary S Friedman. Levofloxacin-Associated Achilles Tendon Rupture. *Ann Pharmacother*; **37**: 1014-1017.

Johannes Beltinger, Manuel Haschke, Priska Kaufmann, Marc Michot, Luigi Terracciano, and Stephan Krähenbühl. Hepatic Veno-Occlusive Disease Associated with Immunosuppressive Cyclophosphamide Dosing and Roxithromycin. *Ann Pharmacother*; **40**: 767-770.

Michelle W McCarthy, and Denise R Kockler. Clopidogrel-Associated Leukopenia. *Ann Pharmacother*; **37**: 216-219.

Natalie Kennie, Tony Antoniou, and Philip Berger .Elevated Creatine Kinase and Myalgia in a Patient Taking Rosiglitazone. *Ann Pharmacother*; **41**: 521-524.

Seyfettin Köklü, Aydin S Köksal, Mehmet Asil, Halil Kiyici, Sahin Çoban, and Mehmet Arhan. Probable Sulbactam/Ampicillin–Associated Prolonged Cholestasis. *Ann Pharmacother*; **38**: 2055-2058.

Jatinder Mohan Chawla, Ravindra Rao, and Rajesh Sagar. Baclofen-Induced Psychosis. *Ann Pharmacother*; **40**: 2071-2073.

Sunita Bond Stenton, Dawn Dalen, and Kerry Wilbur. Myocardial Infarction Associated with Intravenous Immune Globulin. *Ann Pharmacother*; **39**: 2114-2118.

Aydin S Köksal, Seyfettin Köklü, Levent Filik, Nurgül Sasmaz, and Burhan Sahin. Phenyramidol-Associated Liver Toxicity. *Ann Pharmacother*; **37**: 1244-1246.
